# The FEAR Protein Slk19 Restricts Cdc14 Phosphatase to the Nucleus until the End of Anaphase, Regulating Its Participation in Mitotic Exit in *Saccharomyces cerevisiae*

**DOI:** 10.1371/journal.pone.0073194

**Published:** 2013-09-10

**Authors:** Ann Marie E. Faust, Catherine C. L. Wong, John R. Yates III, David G. Drubin, Georjana Barnes

**Affiliations:** 1 Department of Molecular and Cell Biology, University of California, Berkeley, California, United States of America; 2 Department of Chemical Physiology, The Scripps Research Institute, La Jolla, California, United States of America; University of Minnesota, United States of America

## Abstract

In *Saccharomyces cerevisiae* mitosis, the protein Slk19 plays an important role in the initial release of Cdc14 phosphatase from the nucleolus to the nucleus in early anaphase, an event that is critical for proper anaphase progression. A role for Slk19 in later mitotic stages of Cdc14 regulation, however, has not been demonstrated. While investigating the role of Slk19 post-translational modification on Cdc14 regulation, we found that a triple point mutant of *SLK19*, *slk19^3R^* (three lysine-to-arginine mutations), strongly affects Cdc14 localization during late anaphase and mitotic exit. Using fluorescence live-cell microscopy, we found that, similar to *slk19Δ* cells, *slk19^3R^* cells exhibit no defect in spindle stability and only a mild defect in spindle elongation dynamics. Unlike *slk19Δ*cells, however, *slk19^3R^* cells exhibit no defect in Cdc14 release from the nucleolus to the nucleus. Instead, *slk19^3R^* cells are defective in the timing of Cdc14 movement from the nucleus to the cytoplasm at the end of anaphase. This mutant has a novel phenotype: *slk19^3R^* causes premature Cdc14 movement to the cytoplasm prior to, rather than concomitant with, spindle disassembly. One consequence of this premature Cdc14 movement is the inappropriate activation of the mitotic exit network, made evident by the fact that *slk19^3R^* partially rescues a mutant of the mitotic exit network kinase Cdc15. In conclusion, in addition to its role in regulating Cdc14 release from the nucleolus to the nucleus, we found that Slk19 is also important for regulating Cdc14 movement from the nucleus to the cytoplasm at the end of anaphase.

## Introduction

In eukaryotic cells, mitosis occurs in tightly coordinated stages to ensure high-fidelity chromosome segregation. Much of this regulation is controlled by cyclin-dependent kinases (CDKs) and opposing phosphatases. In *Saccharomyces cerevisiae*, the sole CDK, Cdc28, is primarily opposed by the phosphatase Cdc14 during mitosis [1]. The balance between Cdc28 and Cdc14 activities allows the microtubule-based spindle to elongate and the chromosomes to separate during anaphase [2] and mediates mitotic exit and cytokinesis [3]. Because *S. cerevisiae* undergoes a closed mitosis, a major mechanism regulating kinase and phosphatase activities is the control of their subcellular localization throughout the cell cycle.

In *S. cerevisiae*, Cdc14 activity is mainly controlled in two stages during mitosis: by the FEAR (CdcFourteen Early Anaphase Release) network during early anaphase and by the MEN (Mitotic Exit Network) during late anaphase and telophase [4]. Prior to anaphase onset, Cdc14 is sequestered in the nucleolus by its binding partner, Net1, and is considered inactive [5]. After anaphase onset, Esp1 (separase), Slk19 and Spo12, members of the FEAR pathway, promote the partial release of Cdc14 from the nucleolus to the nucleus [6], where it dephosphorylates Cdc28-phosphorylated nuclear substrates [7], [8], [9], [10]. At the end of anaphase, MEN proteins (Tem1, Lte1, Bub2, Bfa1, Cdc15, Dbf2, Dbf20, Mob1 and Cdc5) promote the further release of Cdc14 from the nucleus to the cytoplasm, where it participates in the MEN to resolve and complete mitosis [1].

While the proteins that make up the FEAR and MEN pathways have been identified, their specific roles in Cdc14 regulation and the interactions between the FEAR and MEN networks are less well understood. It was recently shown that the MEN kinase Dbf2 directly phosphorylates Cdc14 and promotes its release from the nucleus to the cytoplasm [11] and that Cdc14 dephosphorylates Mob1, the binding partner of Dbf2, as part of a MEN feedback loop [12]. In the FEAR pathway, Esp1 (separase) and its binding partner Slk19 facilitate the initial release of Cdc14 from the nucleolus to the nucleus, and full or partial deletion of *SLK19* results in a defect in Cdc14 release [6]. However, it is not known whether FEAR proteins, including Slk19, affect Cdc14 localization during later stages of anaphase or influence mitotic exit.

Slk19 appears to be a multi-functional mitotic protein: it has established roles in both FEAR activity and spindle dynamics. Mutants of *SLK19* are synthetic lethal with mutants of *KAR3*, which encodes a minus-end-directed microtubule motor protein, indicating a role for Slk19 in spindle dynamics. In *S. cerevisiae*, deletion of *SLK19* causes shortened mitotic spindles [13]. The metazoan homolog of Slk19, CENP-F (Mitosin), also plays roles in spindle stability, chromosome segregation and the metaphase spindle checkpoint [14]. With regard to FEAR activity, one study found that Slk19 physically interacts with Esp1, and both proteins participate in the FEAR network to release Cdc14 from the nucleolus to the nucleus [15]. A *slk19Δ* mutant, however, exhibits defects in both processes (spindle stability and Cdc14 nucleolus release), which occur during the same cell cycle stage, making it difficult to analyze the role of Slk19 in each process. A recent *SLK19* gene truncation analysis suggested that these two major functions of Slk19 are independent [16]. These functions might be distinguished by differences in the post-translational modification state of Slk19, its protein binding partners or its localization pattern throughout the cell cycle. In this study, we aimed to gain a greater understanding of the role of Slk19 in anaphase progression by identifying *slk19* mutants that can be distinguished from *slk19Δ* mutants by their unique effects on spindle dynamics or FEAR activity.

We generated a combination point mutant of *SLK19*, *slk19^3R^*, that allowed us to identify a novel role for Slk19 in anaphase regulation. While *slk19Δ* cells exhibit a defect in Cdc14 release from the nucleolus to the nucleus in early anaphase (FEAR activity), obscuring the investigation of later stages of Cdc14 regulation, *slk19^3R^* cells have no such FEAR defect and allow normal movement of Cdc14 from the nucleolus to the nucleus. Unexpectedly, in these *slk19^3R^* cells, Cdc14 moves to the cytoplasm prematurely with respect to spindle disassembly. This premature movement of Cdc14 appears to allow the activation of the MEN, as *slk19^3R^* is able to partially rescue the late anaphase arrest phenotype of a mutant of the MEN kinase Cdc15. Therefore, Slk19 appears to play a novel role in anaphase regulation by restricting Cdc14 to the nucleus until mitotic exit and preventing inappropriate exit from mitosis.

## Materials and Methods

### Yeast strains and media

The strains used for all experiments were derivatives of S288C. Strains and genotypes are listed in Table S1 in File S1. Yeast were grown in standard rich medium (YPD) at 30°C for mutagenesis, immunoprecipitation and protein expression experiments.

### Generation of *SLK19* point mutants

To generate missense point mutations in the *SLK19* coding sequence, the entire *SLK19* gene was amplified by PCR from wild type genomic DNA and subcloned into a pBluescript vector, generating pAFSLK19. A 3xHA: *KanMX6* or 13xMyc: *HIS3* epitope tag, derived from plasmids described previously [17], was inserted in-frame with the *SLK19* gene into pAFSLK19. Single base pairs were mutated in pAFSLK19 using the QuikChange Mutagenesis kit (Qiagen). Point mutations were verified by DNA sequencing. The mutated *slk19* gene sequences were amplified by PCR, purified and transformed directly into wild type yeast. Putative genomic integrants were identified by selection on YPD agar plates containing geneticin (Gibco) or agar plates with synthetic minimal medium lacking histidine. These selections were followed by DNA sequencing of transformants and expression analyses of epitope-tagged Slk19 proteins by western blot. The mutant DNA sequences were deposited into GenBank under accession numbers KF285462-KF285469.

### Cell extract preparation and immunoblotting

Equal optical density (OD_600_) equivalents of yeast cells were centrifuged and snap frozen in liquid nitrogen. The pellets were thawed and resuspended in 15% trichloroacetic acid (TCA) in Tris-EDTA. Glass beads were added to the microcentrifuge tubes, after the cell lysates were shaken in a microtube mixer (MT-360; Tomy) at 4°C for 15 minutes. After centrifugation, the supernatant was removed from the beads, transferred into a fresh microcentrifuge tube and centrifuged again at 14,000 rpm for 10 minutes at 4°C. The resulting supernatant was removed by aspiration, and the pellets were washed with cold 100% acetone (−20°C). The pellets were air dried and resuspended in 2X protein sample buffer (20% glycerol, 4% SDS, 5% β-mercaptoethanol, 0.001% bromophenol blue). After incubation at 70°C for 10 minutes, the samples were loaded onto 7.5% acrylamide gels for sodium dodecyl sulfate polyacrylamide gel electrophoresis (SDS-PAGE). The proteins were transferred to nitrocellulose membranes, blocked with TBS-T/5% skim milk and incubated with α-HA antibody (12CA5 mouse monoclonal, 1:5,000, Roche) or α-Pgk1 antibody (22C5 mouse monoclonal, 1:10,000; Molecular Probes) for one hour at room temperature. The blots were washed with TBS-T for 45 minutes and incubated with horseradish peroxidase-conjugated anti-mouse secondary antibody (1:10,000; GE Healthcare) for 45 minutes at room temperature. After washing the blots again with TBS-T for 45 minutes, protein bands were visualized with SuperSignal West ECL reagents (Thermo Scientific).

### Live cell microscopy

For standard microscopy experiments, cultures were grown in imaging medium (synthetic minimal medium containing 2% glucose and required amino acids minus tryptophan) at 25°C until they reached mid-log phase. Cells were pelleted and resuspended in 100 μL of imaging medium. Imaging chambers were prepared by adding 0.2 mg/mL concanavalin A (Con A) to round glass cover slips and incubating at room temperature for 10 minutes. The cover slips were washed with imaging medium, and 50 μL of concentrated cell suspension was spotted onto each cover slip. The cover slips were incubated for 10 minutes to allow cell adherence and then mounted in a custom ring apparatus, the chamber of which was then filled with imaging medium. Cells were imaged using a fluorescence microscope (Olympus IX81) connected to a digital camera at 2-minute intervals, taking 9 z-slices at 0.3-μm intervals for each time point. The Metamorph (Molecular Devices) and ImageJ (http://rsbweb.nih.gov/ij/) software programs were used for image capture and analysis, respectively.

For temperature-sensitive live-cell microscopy experiments, cells were grown to mid-log phase in imaging medium at 25°C. The cells were then shifted to the restrictive temperature (30°C or 37°C) for two hours. After the temperature shift, the cells were processed as described previously, using imaging medium at the appropriate temperature (30°C or 37°C). The cells were imaged on a fluorescence microscope fitted with a temperature-controlled imaging chamber (Olympus IX81-OMAC), and images were captured and analyzed as described above.

### Live cell microscopy imaging measurements

All measurements of live cell images were taken using the ImageJ software program. Spindle and Slk19-GFP spread measurements were taken from still frames of 60-minute movies (2 minutes between frames) using the straight line tool (for straight spindles and lines) or the freeform line tool (for bent spindles and lines). The point of spindle disassembly was taken as the first frame in which the spindle was visibly broken into two segments. Each maximum spindle length measurement was taken in the last frame before spindle disassembly occurred. The appearance of Cdc14-GFP at the bud neck was taken as the first frame in which bud neck fluorescence was visible. For *cdc15-2* disassembly measurements, the disassembly delay time was taken as the time between the frame in which the spindle reached opposite ends of the mother and bud cells and the frame in which the spindle disassembled. At least five spindles were measured for each condition in each experiment.

### Statistical Analysis

Continuous data were analyzed for statistical significance using the Student's t-test. Categorical data were analyzed using Fisher's Exact Test. The p-value for statistical significance was set at p<0.05, with the exception of Slk19-TAP (tandem affinity purification) mass spectrometric enrichment data, where statistical significance was set at p<1×10^−10^.

## Results

### Growth and expression phenotypes of Slk19 mutants

**Figure 1:**
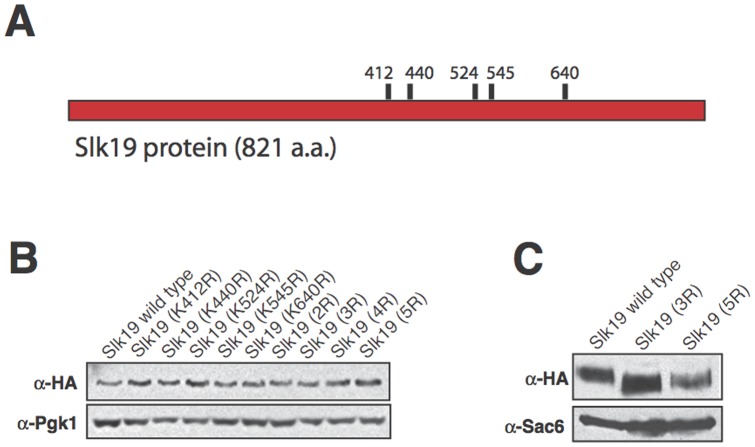
SLK19 point mutants and protein characteristics. Slk19 proteins encoded by specific combination *slk19* point mutants have smaller apparent sizes than wild type Slk19. (A) Diagram of the locations of the *SLK19* point mutations generated in this study. (B) Western blot of Slk19-3xHA proteins from whole-cell extracts, run on an 8.0% polyacrylamide gel. Blot probed with α-HA and α-Pgk1 (loading control) antibodies. Lanes, from left: Slk19 WT; Slk19 (K412R); Slk19 (K440R); Slk19 (K524R); Slk19 (K545R); Slk19 (K640R); Slk19 (K412R, K440R) (2R); Slk19 (K412R, K440R, K524R) (3R); Slk19 (K412R, K440R, K524R, K545R) (4R); Slk19 (K412R, K440R, K524R, K545R, K640R) (5R). (C) Western blot of wild type Slk19-3xHA, Slk19^3R^-3xHA and Slk19^5R^-3xHA proteins from whole-cell extracts, run on a 4.5% polyacrylamide gel to enhance resolution. Blot probed with α-HA and α-Sac6 (loading control) antibodies.

To understand whether Slk19 has separable functions during anaphase, we began by analyzing the effect of the post-translational modification of Slk19 on anaphase progression. Sumoylation is a modification similar to ubiquitination that involves the covalent linkage of the SUMO protein (Smt3 in *S. cerevisiae*) to substrate lysine residues. Sumoylation is thought to occur primarily in the nucleus [18] and therefore could be an important regulatory mechanism for mitosis. Five lysine residues of Slk19 were identified as residues likely to be sumoylated based on an optimized sumoylation clustering consensus sequence, [IVQM][K][X][DE] (H. Aldaz, unpublished data), which is a modification of the published SUMO consensus sequence, [ILV][K][X][DE] [19] (Figure 1A). These five lysine residues were mutated to arginine residues individually and in combination to prevent their sumoylation, and the mutant phenotypes were characterized in terms of cell growth and protein expression.

None of the single or combination point mutants caused bulk growth defects (data not shown). No single point mutant caused a change in protein mobility by SDS-PAGE or in protein expression level relative to wild type Slk19 (Figure 1B). However, the protein encoded by the *slk19* mutant with the consensus lysine residues K412, K440 and K524 mutated to arginine residues (Slk19^3R^) and the protein encoded by the *slk19* mutant with all five consensus lysine residues mutated to arginine residues (Slk19^5R^) had slightly lower apparent molecular weights than wild type Slk19 (approximately 145 kDA for wild type Slk19 vs. 140 kDA for the two mutant proteins; Figure 1C). Slk19^5R^ had a protein expression level that was approximately 50% of the expression levels of either wild type Slk19 or Slk19^3R^. Further investigation showed that Slk19^5R^ and Slk19^3R^ had identical phenotypes. To prevent potential effects of decreased protein expression from complicating the phenotypic analyses, all subsequent experiments were performed with Slk19^3R^.

In addition to assessing Slk19^3R^ and Slk19^5R^ mutant protein expression levels, the sumoylation status of Slk19 was assessed by immunoprecipitation and immunoblotting (File S1). Although our initial findings indicated Slk19 sumoylation, this modification could not be conclusively demonstrated using currently available techniques. Upon immunoprecipitation and immunoblotting, wild type Slk19, Slk19^3R^ and Slk19^5R^ had the same protein band patterns when probed with an anti-Smt3 antibody, and these patterns were not significantly different from the protein band patterns of a control yeast strain (Figure S1 in File S1).

We then assessed the sumoylation status of Slk19 directly by mass spectrometry (File S1). Wild type Slk19-TAP was purified from yeast and assessed using mass spectrometry. The Smt3 peptide EQIGG (glutamic acid-glutamine-isoleucine-glycine-glycine), which is the C-terminal fragment of Smt3 that remains covalently linked to target lysine residues after digestion with trypsin, was not detected on any of the five consensus lysine residues (data not shown). These findings indicate that either the mutated lysines of Slk19^3R^ and Slk19^5R^ are not sumoylated in vivo or that their sumoylation occurs in a narrow window of time during the cell cycle, preventing the detection of the sumoylation signal by immunoprecipitation and immunoblot or by mass spectrometry of Slk19 purified from asynchronous yeast cultures.

### Slk19^3R^ has mild defects in spindle elongation dynamics

After assessing the growth and expression characteristics of the *slk19^3R^* triple point mutant, we sought to understand the effects of these mutations on the known functions of Slk19. We first analyzed the protein localization pattern of Slk19^3R^ compared to wild type Slk19. Wild type Slk19 localizes to the kinetochores prior to the activation of the Anaphase-Promoting Complex/Cyclosome (APC/C); upon activation of the APC/C, Esp1, a binding partner of Slk19, cleaves Slk19 between amino acids 77 and 78, and the C-terminal fragment moves to a focused region of the spindle midzone [20]. Thus, during anaphase, Slk19 is present at both the kinetochores and the spindle midzone. In mid-to-late anaphase, prior to spindle disassembly, the midzone localization of Slk19 disappears, leaving two Slk19 foci, one at each kinetochore cluster.

**Figure 2:**
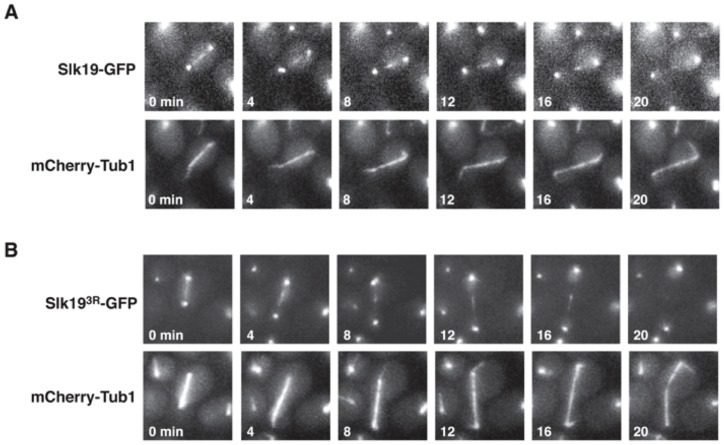
Slk19^3R^ has an altered localization pattern during mitosis. Live cell microscopy time course montages of the endogenous fusion proteins (A) Slk19-GFP and (B) Slk19^3R^-GFP during anaphase in representative cells. Microtubules are visualized with mCherry-Tub1. For each time course, the “0 min” time point was set as the frame immediately prior to the anaphase spindle entering the bud.

To assess the protein localization patterns of wild type Slk19 and Slk19^3R^, we created *SLK19-GFP* and *slk19^3R^-GFP* fusion genes that were integrated at the endogenous *SLK19* locus and encoded Slk19-GFP fusion proteins. Wild type Slk19-GFP has a characteristic Slk19 localization pattern (kinetochore and spindle midzone localization). Slk19^3R^-GFP also localizes to the kinetochores and spindle midzone, but its localization at the midzone is aberrant. Instead of being tightly focused at the spindle midzone, the Slk19^3R^-GFP midzone signal is more spread out along the spindle than the wild type Slk19-GFP signal. The average spread on the spindle during mitosis was 0.913 μm for wild-type Slk19-GFP and 1.155 μm for Slk19^3R^-GFP (p<0.0001; Figure 2A and B; Table 1). The average maximum spindle length prior to spindle disassembly of the *slk19^3R^* mutant does not differ from that of *SLK19* (6.45±0.83 μm for *SLK19* vs. 6.63±0.49 μm for *slk19^3R^*; p = 0.336) (Table 1). Furthermore, the average distribution of Ase1, a major spindle midzone protein, at the midzone during anaphase did not differ significantly between *SLK19* and *slk19^3R^* cells (1.12 μm for *SLK19*; 1.05 μm for *slk19^3R^*; p = 0.287), suggesting that the difference in the distribution of Slk19^3R^-GFP at the midzone is not due to a change in the spindle midzone length. These results indicate that Slk19^3R^ has slightly aberrant localization on the spindle midzone that does not affect spindle length.

**Table 1:** Spread of Slk19-GFP species and Ase1-RFP on spindle vs. spindle length prior to spindle disassembly.

Genotype	Average spread of Slk19-GFP species on spindle	Maximum spindle length prior to disassembly (mean ±SD)	Average spread of Ase1-RFP on spindle
*SLK19*	**0.913** μ**m (n = 50)**	**6.45±0.83** μ**m (n = 34)**	**1.12** μ**m**
*slk19^3R^*	**1.155** μ**m** * **(n = 50)**	**6.63±0.49** μ**m^†^ (n = 23)**	**1.05** μ**m^††^**

* P-value <0.0001 vs. *SLK19* (Student's t-test). † P-value 0.336 vs. *SLK19* (Student's t-test). †† P-value 0.287 *vs. SLK19* (Student's t-test).

**Figure 3:**
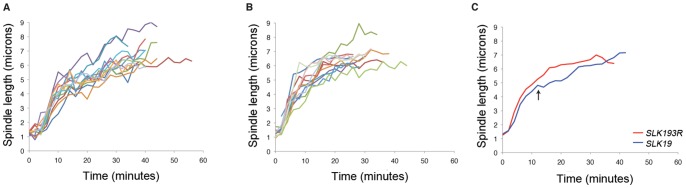
*slk19^3R^* cells lose the transition between the fast and slow phases of anaphase . Anaphase spindle length traces of (A) *SLK19* and (B) *slk19^3R^* are represented by individual colored lines. Each line represents a single spindle (*SLK19*, n = 13; *slk19^3R^*, n = 15). (C) Average spindle elongation traces of *SLK19* (blue) and *slk19^3R^* (red). Each time point represents a minimum of three spindle measurements. Time points with fewer than three spindle measurements were omitted from the average traces. The anaphase pause period is indicated with an arrow. The “0” time point was set at 2 minutes before the beginning of the fast phase of anaphase B.

Slk19 is important for spindle midzone organization, which is critical for proper spindle dynamics [2]. Thus, we sought to understand whether *slk19^3R^* affects spindle dynamics. Unlike the phenotype of *slk19Δ* cells, which have short mitotic spindles [13], *slk19^3R^* cells have mitotic spindles that are of normal length. Similar to the phenotype observed in *slk19Δ* cells [16], *slk19^3R^* spindles lose the transition between the fast and slow phases of spindle elongation during anaphase. The fast phase is thought to represent the initial sliding of the two halves of the short spindle, and the slow phase is thought to represent microtubule polymerization at the plus ends of the two spindle halves [21]. While spindles in *SLK19* cells undergo a distinct “pause” period of 2–4 minutes between the fast and slow phases of anaphase, *slk19^3R^* cells lack this pause period and continuously elongate until they reach their final lengths (Figure 3A–C). This phenotype is identical to that observed previously for *slk19Δ* cells [16]. These findings suggest that Slk19 plays a role in the mid-anaphase pause under certain conditions, but unlike *slk19Δ*, *slk19^3R^* causes aberrant spindle elongation dynamics but no defects in overall spindle length.

### *slk19^3R^* causes defects in Cdc14 localization dynamics

**Figure 4:**
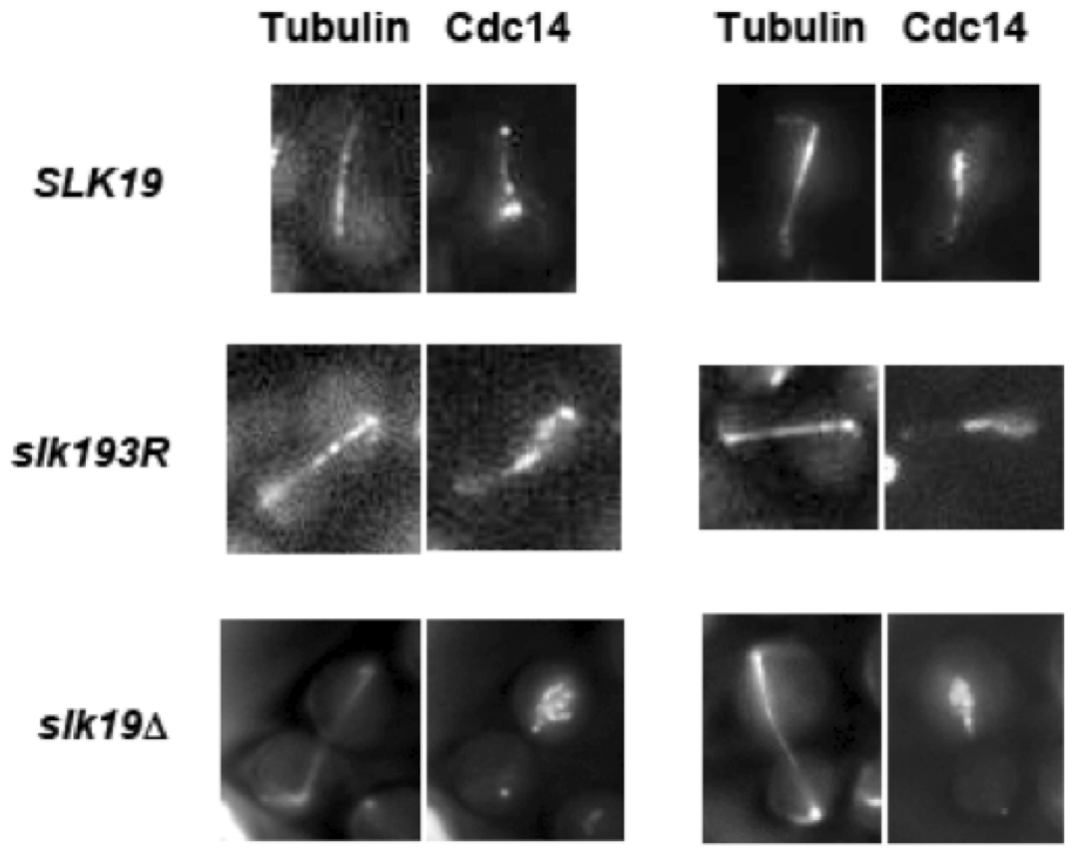
*slk19^3R^* does not cause a defect in Cdc14 nucleolar release during anaphase. Live cell microscopy images of representative cells with Cdc14-GFP and mCherry-Tub1 with *SLK19*, *slk19^3R^* and *slk19Δ* genotypes. In *SLK19* and *slk19^3R^* cells, Cdc14 is released into the nucleus in early-to-mid anaphase (spindle lengths in *SLK19*: 5.5 and 4.9 μM; spindle lengths in *slk19^3R^*: 5.7 and 5.2 μM), while in *slk19Δ*cells, Cdc14 is still sequestered in the nucleolus in late anaphase (spindle lengths in *slk19Δ*: 7.9 and 6.6 μM).

**Figure 5:**
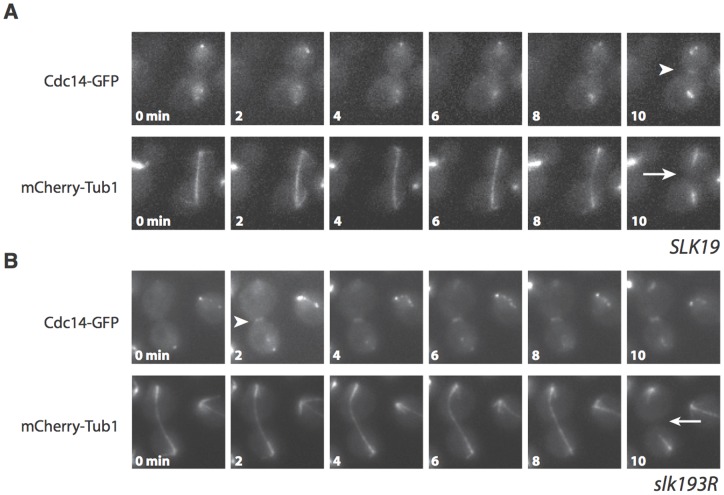
*slk19^3R^* causes premature movement of Cdc14 from the nucleus to the cytoplasm at the end of anaphase. Live cell microscopy time course montages of representative Cdc14-GFP and mCherry-Tub1 in (A) *SLK19* and (B) *slk19^3R^* cells. The appearance of Cdc14-GFP at the bud neck is indicated with arrows, and spindle disassembly is indicated with arrowheads. For the *slk19^3R^* condition in (B), the “0 min” time point was set as the image frame immediately prior to the appearance of Cdc14-GFP at the bud neck. As spindle disassembly occurred during the “10 min” time frame in *slk19^3R^*, a corresponding 10-min time period was depicted in the *SLK19* condition in (A).

Slk19 is involved in spindle dynamics and in the FEAR pathway, which are processes that involve the phosphatase Cdc14. The phosphorylation and dephosphorylation of a number of midzone components by Cdc28 and Cdc14, respectively, regulate spindle dynamics. Because anaphase progression is dependent on these phosphorylation and dephosphorylation events, and because *slk19Δ* mutants are defective in the release of Cdc14 from the nucleolus to the nucleus, we assessed whether *slk19^3R^* also has an effect on Cdc14 localization or activity. First, we investigated whether *slk19^3R^*, like *slk19Δ*, affects the characteristic localization pattern of Cdc14. In *slk19Δ*cells, the release of Cdc14 from the nucleolus to the nucleus is diminished, resulting in a partial release phenotype [6]. We found that, unlike *slk19Δ* cells, *slk19^3R^* cells have no defect in Cdc14 release from the nucleolus to the nucleus (Figure 4). However, *slk19^3R^* cells have a defect in the timing of the second stage of Cdc14 movement, which is the movement of Cdc14 from the nucleus to the cytoplasm at the end of anaphase and consequent localization to the bud neck. In wild type cells, the appearance of Cdc14 at the bud neck is tightly coupled to spindle disassembly because the MEN is responsible for both Cdc14 release to the cytoplasm and mitotic exit [5]. We found that in *SLK19* cells, Cdc14 appearance at the bud neck occurred approximately concomitant with spindle disassembly (1.8±1.6 minutes post-disassembly). In *slk19^3R^* mutants, however, Cdc14 appears at the bud neck several minutes prior to spindle disassembly (5.7±2.6 minutes pre-disassembly), indicating that in *slk19^3R^* cells, Cdc14 release to the cytoplasm occurs prematurely with respect to spindle disassembly (Figure 5A and B; Table 2). Other proteins have been implicated in the regulation of Cdc14 movement from the nucleus to the bud neck, including the MEN kinases Dbf2 [11] and Cdc15 [22] as well as the Polo-like kinase Cdc5 [23]. However, after analyzing the protein-protein interactions of wild type Slk19 purified from yeast by mass spectrometry, we found that none of the MEN proteins or Cdc5 was significantly associated with Slk19 in vivo (Table S2 in File S1). This finding suggests that the role of Slk19 in restricting Cdc14 to the nucleus until the end of anaphase is not dependent on a direct physical interaction with MEN proteins.

**Table 2:** Timing of Cdc14-GFP appearance at the bud neck with respect to spindle disassembly.

Genotype	Average time of Cdc14-GFP appearance at bud neck	P-value* vs. *SLK19*
*SLK19*	**+1.8±1.6 min** (n = 24)	Not applicable
*slk19^3R^*	**−5.7±2.6 min** (n = 15)	<0.0001
*ase1Δ*	**+0.5±3.0 min** (n = 15)	0.104

“+” indicates appearance at the bud neck after spindle disassembly; “−” indicates appearance at the bud neck prior to spindle disassembly. * P-values were determined using the Student's t-test.

This defect in the timing of Cdc14 release to the bud neck could be due to a defect in anaphase spindle dynamics, as our mass spectrometric analysis indicated that a wild type Slk19 associates with a number of spindle-related proteins in vivo (including Kar3, Cik1, Dyn1, Nip100, Stu1, Sli15, Dam1 and Cdc20; Table S2 in File S1), and *slk19^3R^* mutants cause slightly aberrant spindle dynamics. To investigate this possibility, we assessed the movement of Cdc14 from the nucleus to the bud neck in an *ase1Δ* strain, which also has aberrant spindle dynamics; *ase1Δ* cells have fragile spindles that frequently break and are defective in elongation [24]. In *ase1Δ* cells, however, Cdc14 moves from the nucleus to the bud neck with similar timing to that of wild type cells (0.5±3.0 minutes post-disassembly for *ase1Δ*vs. 1.8±1.6 minutes post-disassembly for wild type p = 0.104; Table 2). This finding indicates that Cdc14 movement from the nucleus to the bud neck is decoupled from spindle disassembly in *slk19^3R^* cells and that this premature movement is not due to a general defect in anaphase spindle dynamics.

### *slk19^3R^* partially rescues the phenotype of a temperature-sensitive MEN mutant

**Figure 6:**
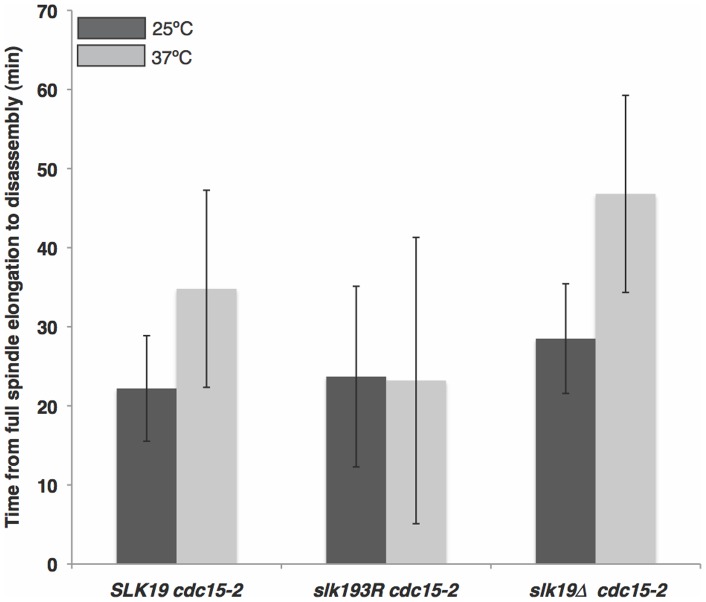
*slk19^3R^* partially rescues the spindle disassembly arrest phenotype of *cdc15-2* cells. Graph represents the delay in spindle disassembly of *SLK19 cdc15-2* (n = 10), *slk19^3R^ cdc15-2* (n = 24) and *slk19Δ cdc15-2* (n = 23) cells at 25°C and *SLK19 cdc15-2* (n = 19), *slk19^3R^ cdc15-2* (n = 23) and *slk19Δ cdc15-2* (n = 18) cells at 37°C. *SLK19* and *slk19Δ* are unable to rescue the *cdc15-2* disassembly defect at the restrictive temperature (p<0.01 for both), but *slk19^3R^* rescues the defect to levels that are not statistically significantly different from those at the permissive temperature.

After determining that *slk19^3R^* causes the premature release of Cdc14 to the cytoplasm, we assessed the effect of such a defect in the timing of Cdc14 localization. Cdc14 is released from the nucleus to the cytoplasm through the actions of MEN proteins; once it reaches the cytoplasm, Cdc14 participates as a member of the MEN to promote mitotic exit [25]. To understand whether premature release of Cdc14 to the cytoplasm has an effect on mitotic exit, we assessed the ability of *slk19^3R^* to suppress the late anaphase arrest phenotype of the temperature-sensitive mutant *cdc15-2*. The essential kinase Cdc15 is an integral component of the MEN. At the restrictive temperature, a *cdc15-2* mutant arrests at the end of anaphase with fully elongated spindles, strongly delaying or preventing spindle disassembly [26]. We compared the spindle disassembly defects (defined as the time between full spindle elongation and spindle disassembly) of *cdc15-2* cells in the genetic backgrounds of *SLK19*, *slk19^3R^* and *slk19Δ*. As shown in Figure 6, *SLK19 cdc15-2* cells were significantly delayed in spindle disassembly at 37°C compared to 25°C (34.8±12.5 minutes at 37°C vs. 22.2±6.7 minutes at 25°C; p<0.01). However, this defect was rescued in *slk19^3R^ cdc15-2* cells, which underwent spindle disassembly with no delay (23.2±18.1 minutes at 37°C vs. 23.7±11.4 minutes at 25°C; p = 0.919); the difference in delay times between *SLK19* and *slk19^3R^* cells is statistically significant (p = 0.02). This rescue of delayed spindle disassembly did not occur in *slk19Δ cdc15-2* cells (46.8±12.5 minutes at 37°C vs. 28.5±6.9 minutes at 25°C; p<0.01), indicating that this effect is not due to a loss of Slk19 protein function. These findings indicate that by allowing the premature movement of Cdc14 to the cytoplasm, *slk19^3R^* allows spindle disassembly to proceed under conditions of decreased MEN function.

**Figure 7:**
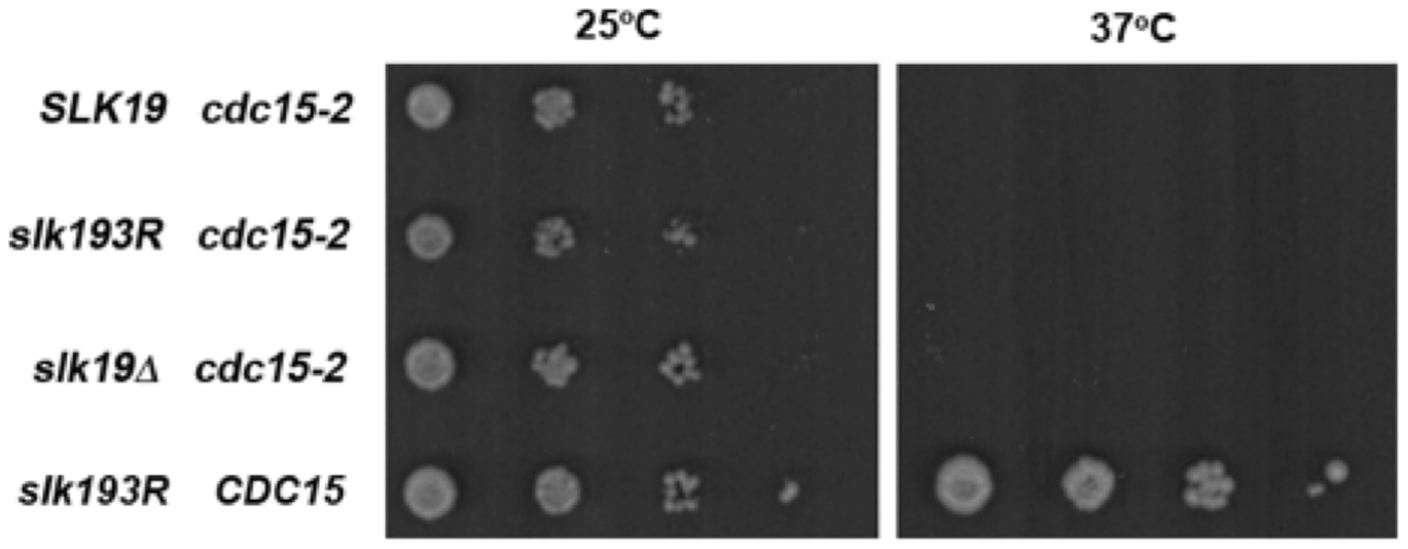
*slk193R* does not rescue the temperature sensitivity phenotype of cdc15-2 cells. The indicated strains were spotted in serial dilutions from 0.5 OD_600_ units/mL to 0.5×10^−3^ OD_600_ units/mL onto YPD plates and grown at 25°C or 37°C for 3 days. Strains with *cdc15-2* and either *SLK19*, *slk19^3R^* or *slk19Δ* formed colonies at 25°C but did not form colonies at 37NC. A strain with *CDC15* and *slk19^3R^* formed colonies at both temperatures.

The effect of *slk19^3R^* on mitotic exit might indicate that *slk19^3R^* is a gain-of-function mutant. If this were the case, then *slk19^3R^* would be expected to partially or completely rescue a temperature-sensitive mutation in a mitotic exit mutant. To test this possibility, we grew strains with a temperature-sensitive *cdc15-2* mutation and either *SLK19*, *slk19^3R^* or *slk19Δ* alleles. These strains were plated onto YPD plates as serial dilutions and grown at 25°C (permissive temperature for *cdc15-2*) or 37°C (restrictive temperature for *cdc15-2*). As shown in Figure 7, none of the *cdc15-2* strains grew at 37°C. Only the control strain with wild-type *SLK19* and *CDC15* grew at the restrictive temperature, indicating that *slk19^3R^* is not a gain-of-function mutant.

## Discussion

**Figure 8:**
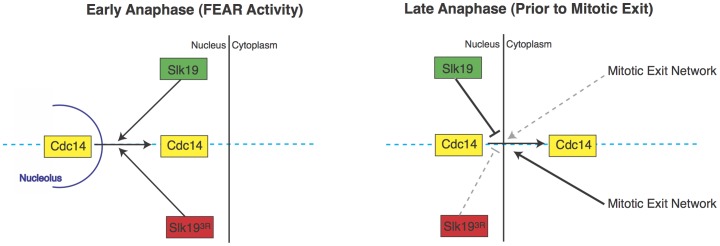
Model of the proposed activities of Slk19 and Slk19^3R^ during anaphase with respect to the regulation of Cdc14 localization. In early anaphase, the FEAR network is activated to release Cdc14 from the nucleolus to the nucleus, and both Slk19 and Slk19^3R^ are proficient at promoting the release of Cdc14 from the nucleolus (diagram, left side). In late anaphase, wild type Slk19 retains Cdc14 within the nucleus until after spindle disassembly, while Slk19^3R^ cannot retain Cdc14 within the nucleus, resulting in the premature release of Cdc14 to the bud neck in the cytoplasm prior to spindle disassembly (diagram, right side). MEN proteins, which are involved in the release of Cdc14 from the nucleus to the bud neck during mitotic exit, might directly oppose Slk19 in late anaphase and might be more effective at promoting the release of Cdc14 to the bud neck in the presence of the mutant Slk19^3R^ protein. Alternatively, Slk19 might retain Cdc14 in the nucleus by a mechanism that not indirect opposition of the MEN, and mutant Slk19^3R^ protein is unable to retain Cdc14 within the nucleus until the end of anaphase.

In this study, we sought to gain a better understanding of the roles of Slk19 in mitosis by investigating the phenotypes of *slk19* point mutants compared to *SLK19* and *slk19Δ*alleles. As a component of the FEAR network, Slk19 plays an important role in the early anaphase release of Cdc14 phosphatase from the nucleolus to the nucleus [6], but its function in other aspects of Cdc14 regulation was unknown. Because *slk19*loss-of-function mutants cause a defect in Cdc14 release from the nucleolus, analyses of the function of Slk19 in later stages of Cdc14 regulation have been challenging. To address this problem, we generated a triple point mutant of *SLK19* that does not prevent its FEAR activity, but causes a specific defect in the regulation of Cdc14 movement from the nucleus to the cytoplasm during late anaphase, uncovering a novel role for Slk19 in late anaphase Cdc14 regulation and mitotic exit (Figure 8).

The primary defect of the *slk19^3R^* point mutant appears to be an inability to restrict Cdc14 to the nucleus until just after spindle disassembly in late anaphase. Slk19^3R^-GFP localizes to a wider region of the anaphase spindle midzone than does Slk19-GFP, but this difference in localization does not result in decreased spindle stability. In addition, this mutant is not defective in FEAR activity, as Cdc14 successfully leaves the nucleolus and enters the nucleus during early anaphase. These results indicate that *slk19^3R^* is not simply a loss-of-function mutant. Unexpectedly, in these mutant cells, Cdc14 appears in the cytoplasm during late anaphase, several minutes prior to spindle disassembly, indicating that *slk19^3R^* causes the uncoupling of Cdc14 release from the nucleus and spindle disassembly. It is formally possible that *slk19^3R^* does not affect the release of Cdc14 from the nucleus but instead affects its ability to localize to the bud neck after nuclear release, but this mechanism of regulation is unlikely, as will be discussed later. We also found that *slk19^3R^* can suppress the late anaphase spindle disassembly arrest phenotype of a *cdc15-2* mutant, indicating that *slk19^3R^* can drive mitotic exit even when Cdc15 function is diminished or absent.

In wild type cells, Cdc14 appears at the bud neck in the cytoplasm in a manner that is coupled to spindle disassembly, owing to the fact that both Cdc14 release from the nucleus and spindle disassembly are controlled by the MEN [25], [27]. Cdc14 is known to dephosphorylate two components of the MEN, the kinase Cdc15 and the Dbf2 kinase partner Mob1 [28], [12]. In turn, the kinase activities of Dbf2 and Cdc15 are involved in the release of Cdc14 to the cytoplasm [5], [29], [11]. These phosphorylation and dephosphorylation events are part of a positive feedback loop in mitotic exit. While this feedback loop seems to be important for mitotic exit, the dominant effector of the loop is unclear because of the inter-relationships among the proteins involved. The results of the present study, wherein premature movement of Cdc14 to the cytoplasm correlates with the rescue of a *cdc15-2* defect in spindle disassembly, suggest a dominant role for Cdc14 in this feedback loop. This information, summarized in the model in Figure 8, suggests that Slk19 plays a critical role in retaining Cdc14 in the nucleus during anaphase, preventing the MEN-mediated release of Cdc14 to the cytoplasm and consequent activation of this feedback loop until the appropriate point in the cell cycle. A decrease in this Slk19 function due to the *slk19^3R^* mutation allows Cdc14 to move to the cytoplasm prematurely and activate this mitotic exit feedback loop under conditions of decreased MEN function.

In addition to demonstrating the effects of inappropriate movement of Cdc14 to the cytoplasm, this study introduces a novel mechanism of cross talk between components of the FEAR pathway and mitotic exit. Previous studies have shown that the Polo-like kinase Cdc5 plays roles in both the FEAR and MEN pathways [23], [30], [31]. Other studies have shown that the MEN kinase Cdc15 is activated through dephosphorylation by Cdc14 during anaphase [32], [28]. Prior to our present study, however, there was no known role for Slk19 in the regulation of mitotic exit. Our results indicate a broader role for Slk19-mediated regulation of Cdc14 localization and additional complexity in the relationship between the FEAR pathway and mitotic exit.

In the present study, we demonstrated that the *slk19^3R^* mutant has a defect in retaining Cdc14 in the nucleus at the end of anaphase, but the mechanism of why this point mutant possesses this defect is unclear. Several possible mechanisms, discussed below, might account for the observed Slk19^3R^ phenotype. First, Slk19 might normally cooperate with its FEAR binding partner Esp1 to retain Cdc14 in the nucleus until mitotic exit, and Slk19^3R^ might be unable to properly interact with Esp1. However, Esp1 leaves the spindle midzone significantly earlier in anaphase than Slk19 [2], and the physical interaction between Slk19 and Esp1 is diminished during anaphase and mitotic exit [33], so it is unlikely that Esp1 participates in this late anaphase function of Slk19.

Another possibility to explain how Slk19 affects Cdc14 localization is through an interaction between Slk19 and one or more components of the MEN, which mediate the release of Cdc14 from the nucleus to the bud neck at the end of anaphase. Slk19^3R^ protein might be unable to take part in this interaction, which would allow MEN components to mediate the premature release of Cdc14 to the cytoplasm. However, our mass spectrometry analysis did not show a specific association between Slk19 and any MEN components in vivo, suggesting that this effect is unlikely to be due to a direct protein-protein interaction. Alternatively, Slk19^3R^ might bypass the requirement for MEN-mediated Cdc14 release from the nucleus to the cytoplasm, acting in a dominant negative manner. If this were the case, then in the presence of Slk19^3R^, the loss of MEN components would have little or no effect on Cdc14 movement to the bud neck at the end of anaphase. If MEN activity were still required for Cdc14 release to the cytoplasm, then mutations in or the loss of MEN components would be epistatic to *slk19^3R^*.

A third possible mechanism for the observed phenotype of *slk19^3R^* is that Slk19^3R^ protein has an altered phosphorylation pattern, promoting premature mitotic progression. Treatment of Slk19 and Slk19^3R^ with lambda phosphatase indicates that both proteins are phosphorylated, as both proteins have similar decreases in apparent molecular weight after phosphatase treatment (data not shown). A mass spectrometric analysis to detect phosphorylated residues at different points in the cell cycle would be necessary to further understand the phosphorylation status of Slk19 and Slk19^3R^.

It is formally possible that Slk19 physically associates with Cdc14 and regulates its accumulation at the bud neck but not its release from the nucleus. In this scenario, Slk19^3R^ would be deficient in this interaction, allowing Cdc14 to accumulate at the bud neck prematurely with respect to spindle disassembly. This mechanism is unlikely, however, for two reasons. First, we performed a TAP purification of Slk19 to identify physical protein-protein interactions and found no physical interaction between Slk19 and Cdc14 (Table S2 in File S1). Second, the present study and several previous studies have investigated the subcellular localization of Slk19 [2], [13], [20], and Slk19 has an exclusively nuclear localization pattern.

Another possible mechanism of regulation is that Slk19 affects some aspect of the mitotic spindle, which interacts with the bud neck and affects the accumulation of Cdc14 at the bud neck. Slk19 interacts with many other mitotic spindle-associated proteins, so it is possible that *slk19^3R^* has some effect on the structure or composition of the mitotic spindle. However, the mitotic spindle is localized to the nucleus, and bud neck components are generally thought to be cytoplasmic, so it is unclear how these two structures could interact. Further work would be required to determine whether *slk19^3R^* causes altered bud neck composition or organization.

A final possible mechanism for the role of Slk19 in retaining Cdc14 in the nucleus is that sumoylation of Slk19 might be responsible for its ability to affect Cdc14 localization. While we were unable to detect sumoylation of total Slk19 protein on the targeted SUMO consensus lysines in a population of cells or of total Slk19 sumoylation in vivo, it remains a possibility that we did not detect it because one or more of these consensus lysines are sumoylated transiently in only a narrow window of time during mitosis, allowing Slk19 to regulate Cdc14 movement between the nucleus and the cytoplasm. Future studies involving finely tuned cell synchronization and sumoylation detection assays will be necessary to determine whether Slk19 is sumoylated in vivo and whether the sumoylation status of Slk19 affects Cdc14 localization.

Why *slk19^3R^* cells lose the short anaphase pause during spindle elongation in early-to-mid anaphase is unclear, but because this phenotype is also observed in *slk19Δ*cells, it is probably related to a general loss of function. Because Cdc14 activity is linked to spindle elongation [2], this phenotype could be caused by defects in Cdc14 activity due to the loss of Slk19-regulated Cdc14 localization. It would be interesting to know whether Cdc14 phosphatase activity is affected in a *slk19^3R^* mutant and whether spindle-associated Cdc14 target proteins have altered phosphorylation patterns or altered functions in this mutant background.

In conclusion, we have used a specific set of point mutations in *SLK19* to identify a novel activity of Slk19 in Cdc14 regulation. We bypassed the Cdc14 early anaphase release defect of *slk19Δ* cells and have shown that Slk19, in addition to facilitating Cdc14 release from the nucleolus to the nucleus during early anaphase, also restricts the localization of Cdc14 to the nucleus until the end of anaphase. This restricted localization of Cdc14 by Slk19 limits MEN activity to the proper temporal point in mitosis. These findings suggest a novel form of cross talk between the FEAR pathway and mitotic exit and a novel mechanism of regulation of Cdc14 phosphatase activity that is mediated by Slk19.

## Supporting Information

File S1 (DOCX)
