# Supplementary material for: The FEAR Protein Slk19 Restricts Cdc14 Phosphatase to the Nucleus until the End of Anaphase, Regulating Its Participation in Mitotic Exit in Saccharomyces cerevisiae
Source: PLoS One. 2013 Sep 10;8(9):e73194. doi: 10.1371/journal.pone.0073194 (PMC3769316; doi:10.1371/journal.pone.0073194)
Supplement: File S1 — (DOCX) [file pone.0073194.s001.docx]

**FILE S1 – SUPPORTING INFORMATION**

Figure S1 Slk19 sumoylation pattern is not altered in Slk19^3R^ and Slk19^5R^ mutant proteins. Western blots of Ndc10-13xMYC, DDY 904 (negative control) cells, Slk19-13xMyc, Slk19^3R^-13xMYC and Slk19^5R^-13xMYC immunoprecipitated with α-MYC antibody. Top blot shows immunoprecipitated proteins visualized with α-Smt3 antibody. Bottom blot shows MYC input lanes.

**Supporting Materials and Methods**

**Immunoprecipitation and immunoblotting of sumoylated proteins**

**Immunoprecipitation**: Cells were grown under standard conditions, washed with ddH_2_O, snap frozen in liquid nitrogen and stored at -80^°^C. Myc immunoprecipitation experiments were performed as described previously [1] with some modifications. After total protein extracts were prepared, they were precleared for 30 minutes at 4^º^C with protein G Sepharose (40 μL) (GE Healthcare) before incubation with 5 μl of α-MYC antibody (9E10; laboratory prep) for one hour at 4°C. After this incubation period, 50 μl of protein G Sepharose was added to each sample, and the suspensions were incubated for 30 minutes at 4°C. The Sepharose beads were washed three times with cold lysis buffer.

**Immunoblotting:** Excess liquid was removed from the protein-conjugated Separhose beads, 30 μl of 2X protein sample buffer (20% glycerol, 4% SDS, 5% β-mercaptoethanol, 0.001% bromophenol blue) was added, and the samples were incubated at 70°C for 10 minutes before being loaded onto 7.5% agarose gels for sodium dodecyl sulfate polyacrylamide gel electrophoresis (SDS-PAGE). The proteins were transferred to nitrocellulose membranes, blocked with TBS-T/5% skim milk and incubated with α−Smt3 antibody (rabbit polyclonal, 1:2,000, kindly provided by P. Hieter) or α-MYC antibody (1:10,000) for one hour at room temperature. The blots were washed with TBS-T for 45 minutes and incubated with α-rabbit secondary antibody (1:10,000; GE Healthcare) for 45 minutes at room temperature. After washing the blots again with TBS-T for 45 minutes, protein bands were visualized with SuperSignal West ECL reagents (Thermo Scientific).

**Slk19-TAP mass spectrometry sample preparation and analysis**

**Purification of Slk19-TAP:** Slk19-TAP was purified as described previously [2], with minor modifications. The initial yeast extract supernatant was passed over a CL-B6 resin (Sigma) column instead of a Q Sepharose resin column. For the final step before snap freezing, the protein sample was eluted off of an IgG Sepharose column using 8 M urea buffer (8.0 M urea, 50 mM Tris-HCl, pH 8.8) instead of protein sample buffer. All other steps were as described in [2].

**Digestion**: The protein was reduced by the addition of 500 mM *tris*(2-carboxyethyl)phosphine (TCEP) to a final concentration of 5 mM (incubated at room temperature for 20 minutes), followed by carboxyamidomethylation of cysteines by incubation at room temperature for 30 minutes in the dark with 500 mM iodoacetamide (final concentration 10 mM). The sample was diluted 4-fold (to 2 M), and trypsin (Promega) was added at approximately 1:100 enzyme:substrate ratio (wt:wt) and then incubated at 37°C overnight in the dark. The resulting peptides were dissolved in 90% formic acid to a final concentration of 5%. The sample was stored at -20°C prior to LC-MS/MS analysis.

HPLC conditions: The peptide mixtures were pressure-loaded onto an equilibrated reversed phase (RP) Column. The microcapillary column was constructed by slurry packing approx. 10 cm of C18 material (5 um, 125A, Aqua; Phenomenex, Torrance, CA) into a 100-um fused silica capillary that had been previously pulled to a tip diameter of approx. 5 μm using a Sutter Instruments laser puller (Sutter Manufacturing, Novato, CA). Separations were performed on an Agilent 1100 quaternary HPLC (Agilent, Palo Alto, CA). The HPLC buffer solutions used were as follows: water:acetonitrile:formic acid (95:5:0.1, v:v:v) as buffer A, water:acetonitrile:formic acid (20:80:0.1, v:v:v) as buffer B. The elution gradient was as follows: 10 min of 100% buffer A, a 5-min gradient from 0 to 15% buffer B, a 65-min gradient from 15 to 45% buffer B, a 15-min gradient from 45 to 100% buffer B, and 5 min of 100% buffer B.

**Mass spectrometry conditions:** Data-dependent tandem mass spectrometry (MS/MS) analysis was performed with a LTQ-Orbitrap mass spectrometer (ThermoFisher, San Jose, CA). Peptides eluted from the LC column were directly electrosprayed into the mass spectrometer with the application of a distal 2.5-kV spray voltage. A cycle of one full-scan MS spectrum (m/z 300-1800) was acquired followed by eight MS/MS events, sequentially generated on the first to the eighth most intense ions selected from the full MS spectrum at a 35% normalized collision energy. The number of microscans was one for both MS and MS/MS scans, and the maximum ion injection time was 50 and 100 ms, respectively. The dynamic exclusion settings used were as follows: repeat count, 1; repeat duration, 30 seconds; exclusion list size, 100; and exclusion duration, 180 seconds. MS scan functions and HPLC solvent gradients were controlled by the Xcalibur data system (ThermoFisher).

**Data analysis:** Full MS and tandem mass spectra were extracted from raw files, and the tandem mass spectra were searched against a *Saccharomyces cerevisiae* protein database (database released on December 16, 2005). To accurately estimate peptide probabilities and false discovery rates, we used a decoy database containing the reversed sequences of all of the proteins appended to the target database (*S28*). Tandem mass spectra were matched to sequences using the Sequest (*S29*) or ProLuCID (*S30*) algorithm. Sequest and ProLuCID searches were conducted on an Intel Xeon 80-processor cluster running under the Linux operating system. The peptide mass search tolerance was set to 3 Da for spectra acquired on the LTQ instrument. The mass of the amino acid cysteine was statically modified by +57.02146 Da to take into account the carboxyamidomethylation of the sample, and serine, threonine and tyrosine were treated as differentially modified by +79.9663 Da for phosphorylation. No enzymatic cleavage conditions were imposed on the database search, so the search space included all candidate peptides whose theoretical mass fell within the mass tolerance window, regardless of their tryptic status (*S31*).

The validity of peptide/spectrum matches (PSMs) was assessed in DTASelect (*S32, S33*) using two SEQUEST (*S29*) defined parameters, the cross-correlation score (XCorr), and normalized difference in cross-correlation scores (DeltaCN). The search results were grouped by charge state (+1, +2, +3, and greater than +3) and tryptic status (fully tryptic, half-tryptic, and non-tryptic), resulting in 12 distinct sub-groups. In each one of these sub-groups, the distribution of Xcorr, DeltaCN and DeltaMass values for (a) direct and (b) decoy database PSMs was obtained, then the direct and decoy subsets were separated by discriminant analysis. Full separation of the direct and decoy PSM subsets is not generally possible; therefore, peptide match probabilities were calculated based on a nonparametric fit of the direct and decoy score distributions. A peptide probability of 90% was set as the minimum threshold. The false discovery rate was calculated as the percentage of reverse decoy PSMs among all the PSMs that passed the 90% probability threshold. After this last filtering step, we estimate that both the protein and peptide false discovery rates were reduced to between 0.0% and 0.5%.

**Supporting Literature Cited**

[1] [Montpetit, B](http://www.ncbi.nlm.nih.gov/pubmed?term=%22Montpetit%20B%22%5BAuthor%5D)., [Hazbun, T.R](http://www.ncbi.nlm.nih.gov/pubmed?term=%22Hazbun%20TR%22%5BAuthor%5D)., [Fields, S](http://www.ncbi.nlm.nih.gov/pubmed?term=%22Fields%20S%22%5BAuthor%5D)., and P. Hieter 2006 Sumoylation of the budding yeast kinetochore protein Ndc10 is required for Ndc10 spindle localization and regulation of anaphase spindle elongation. [J Cell Biol.](javascript:AL_get(this,%20'jour',%20'J%20Cell%20Biol.');) **174(5)**: 653-63.

[2] Cheeseman, I. M., Brew, C., Wolyniak, M., Desai, A., Anderson, S., *et al.* 2001 Implication of a novel multiprotein Dam1p complex in outer kinetochore function. J Cell Biol. **155**: 1137–1146.

**Table S1 Yeast strains and genotypes**

| **Strain** | **Genotype** | **Source** |
| --- | --- | --- |
| DDY 904 | Wild type | D. Drubin |
| DDY 3340 | *SLK19-Stag-TEV-ZZ::KanMX6 ura3-52 leu2-3,112 trp1-1(am) prb1-1122 pep4-3 pre1-451* | S. Westermann |
| CPY 55 | *NDC10-13MYC::KanMX6 his3Δ200 ura3-52 leu2-3,112* | Y. Peng |
| AFY 102 | *SLK19-3HA::KanMX6 his3Δ200 ura3-52 leu2-3,112* | This study |
| AFY 103 | *slk19(K412R)-3HA::KanMX6 his3Δ200 ura3-52 leu2-3,112* | This study |
| AFY 104 | *slk19(K440R)-3HA::KanMX6 his3Δ200 ura3-52 leu2-3,112* | This study |
| AFY 105 | *slk19(K524R)-3HA::KanMX6 his3Δ200 ura3-52 leu2-3,112* | This study |
| AFY 106 | *slk19(K545R)-3HA::KanMX6 his3Δ200 ura3-52 leu2-3,112* | This study |
| AFY 107 | *slk19(K640R)-3HA::KanMX6 his3Δ200 ura3-52 leu2-3,112* | This study |
| AFY 108 | *slk19(K412R,K440R)-3HA::KanMX6 his3Δ200 ura3-52 leu2-3,112* | This study |
| AFY 109 | *slk19(K412R,K440R,K524R)-3HA::KanMX6 his3Δ200 ura3-52 leu2-3,112* | This study |
| AFY 110 | *slk19(K412R,K440R,K524R,K545R)-3HA::KanMX6 his3Δ200 ura3-52 leu2-3,112* | This study |
| AFY 111 | *slk19(K412R,K440R,K524R,K545R,K640R)-3HA::KanMX6 his3Δ200 ura3-52 leu2-3,112* | This study |
| AFY 146 | *SLK19-13MYC::HIS3 his3Δ200 ura3-52 leu2-3,112* | This study |
| AFY 147 | *slk19(K412R,K440R,K524R)-13MYC::HIS3 his3Δ200 ura3-52 leu2-3,112* | This study |
| AFY 148 | *SLK19-GFP::HIS3 mCherry-TUB1::URA3 his3Δ200 ura3-52 leu2-3,112* | This study |
| AFY 149 | *slk19(K412R,K440R,K524R)-GFP::HIS3 mCherry-TUB1::URA3 his3Δ200 ura3-52 leu2-3,112* | This study |
| AFY 150 | *SLK19-13MYC::HIS3 mCherry-TUB1::URA3 his3Δ200 ura3-52 leu2-3,112* | This study |
| AFY 151 | *slk19(K412R,K440R,K524R)-13MYC::HIS3 mCherry-TUB1::URA3 his3Δ200 ura3-52 leu2-3,112* | This study |
| AFY 152 | *ASE1-GFP::KanMX6 mCherry-TUB1::URA3 his3Δ200 ura3-52 leu2-3,112* | This study |
| AFY 153 | *SLK19-13MYC::HIS3 CDC14-GFP::KanMX6 mCherry-TUB1::URA3 his3Δ200 ura3-52 leu2-3,112* | This study |
| AFY 154 | *slk19(K412R,K440R,K524R)-13MYC::HIS3 CDC14-GFP::KanMX6 mCherry-TUB1::URA3 his3Δ200 ura3-52 leu2-3,112* | This study |
| AFY 155 | *slk19Δ::URA3 CDC14-GFP::KanMX6 mCherry-TUB1::URA3 his3Δ200 ura3-52 leu2-3,112* | This study |
| AFY 156 | *ase1Δ::URA3 CDC14-GFP::KanMX6 mCherry-TUB1::URA3 his3Δ200 ura3-52 leu2-3,112* | This study |
| AFY 157 | *SLK19-13MYC::HIS3 cdc15-2 GFP-TUB1::URA3 his3Δ200 ura3-52 leu2-3,112* | This study |
| AFY 158 | *slk19(K412R,K440R,K524R)-13MYC::HIS3* *cdc15-2 GFP-TUB1::URA3 his3Δ200 ura3-52 leu2-3,112* | This study |
| AFY 159 | *slk19Δ::URA3 cdc15-2 GFP-TUB1::URA3 his3Δ200 ura3-52 leu2-3,112* | This study |

**All strains are derived from S288C.**

**Table S2 Proteins enriched in Slk19-TAP purification in yeast**

| Protein ID | Slk19-TAP Peptide Spectrum Count | Total Slk19-TAP Spectrum Count* | Peptide Atlas Spectrum Count | Total Peptide Atlas Spectrum Count** | P-value^#^ |
| --- | --- | --- | --- | --- | --- |
| Slk19 | 3878 | 21240 | 25 | 2705360 | 0 |
| Mcm6 | 112 | 21240 | 56 | 2705360 | 1.11E-191 |
| Cat8 | 68 | 21240 | 6 | 2705360 | 8.31E-136 |
| Mrc1 | 40 | 21240 | 5 | 2705360 | 5.60E-79 |
| Scm3 | 39 | 21240 | 4 | 2705360 | 7.31E-78 |
| Doa1 | 59 | 21240 | 207 | 2705360 | 7.00E-66 |
| Spc110 | 35 | 21240 | 32 | 2705360 | 1.73E-55 |
| Tom1 | 48 | 21240 | 156 | 2705360 | 2.84E-55 |
| Smc5 | 26 | 21240 | 2 | 2705360 | 5.73E-53 |
| YBR242W | 33 | 21240 | 29 | 2705360 | 8.89E-53 |
| Cik1 | 24 | 21240 | 0 | 2705360 | 2.53E-51 |
| Gip1 | 22 | 21240 | 0 | 2705360 | 4.16E-47 |
| Vps27 | 35 | 21240 | 78 | 2705360 | 1.73E-45 |
| Boi2 | 25 | 21240 | 10 | 2705360 | 3.36E-45 |
| Csf1 | 21 | 21240 | 0 | 2705360 | 5.33E-45 |
| Hsl1 | 21 | 21240 | 2 | 2705360 | 1.33E-42 |
| Agp2 | 19 | 21240 | 0 | 2705360 | 8.77E-41 |
| Ysw1 | 19 | 21240 | 0 | 2705360 | 8.77E-41 |
| Ecm5 | 21 | 21240 | 5 | 2705360 | 3.38E-40 |
| Sov1 | 59 | 21240 | 663 | 2705360 | 7.44E-40 |
| YKR015C | 18 | 21240 | 0 | 2705360 | 1.12E-38 |
| Ira1 | 20 | 21240 | 5 | 2705360 | 3.50E-38 |
| Bni1 | 25 | 21240 | 30 | 2705360 | 4.86E-38 |
| Smy2 | 23 | 21240 | 19 | 2705360 | 1.26E-37 |
| Jip4 | 19 | 21240 | 3 | 2705360 | 1.32E-37 |
| Axl1 | 17 | 21240 | 0 | 2705360 | 1.44E-36 |
| Inp2 | 17 | 21240 | 0 | 2705360 | 1.44E-36 |
| Ubp13 | 23 | 21240 | 24 | 2705360 | 4.37E-36 |
| Cdc13 | 23 | 21240 | 28 | 2705360 | 5.17E-35 |
| Ira2 | 19 | 21240 | 7 | 2705360 | 5.48E-35 |
| Bud3 | 22 | 21240 | 22 | 2705360 | 7.42E-35 |
| Kkq8 | 16 | 21240 | 0 | 2705360 | 1.85E-34 |
| Mrpl9 | 16 | 21240 | 0 | 2705360 | 1.85E-34 |
| Zip1 | 16 | 21240 | 0 | 2705360 | 1.85E-34 |
| Rpo31 | 52 | 21240 | 614 | 2705360 | 2.31E-34 |
| Spo14 | 17 | 21240 | 3 | 2705360 | 1.61E-33 |
| Jlp2 | 15 | 21240 | 0 | 2705360 | 2.37E-32 |
| Lpe10 | 15 | 21240 | 0 | 2705360 | 2.37E-32 |
| Rev3 | 15 | 21240 | 0 | 2705360 | 2.37E-32 |
| YDR336W | 15 | 21240 | 0 | 2705360 | 2.37E-32 |
| YPL107W | 15 | 21240 | 0 | 2705360 | 2.37E-32 |
| YPR117W | 15 | 21240 | 0 | 2705360 | 2.37E-32 |
| Tod6 | 17 | 21240 | 7 | 2705360 | 4.74E-31 |
| Ubx2 | 18 | 21240 | 12 | 2705360 | 8.90E-31 |
| Rkm2 | 14 | 21240 | 0 | 2705360 | 3.04E-30 |
| Srd1 | 14 | 21240 | 0 | 2705360 | 3.04E-30 |
| YOR287C | 14 | 21240 | 0 | 2705360 | 3.04E-30 |
| Arg81 | 16 | 21240 | 8 | 2705360 | 1.28E-28 |
| Rga2 | 16 | 21240 | 8 | 2705360 | 1.28E-28 |
| Gcn5 | 26 | 21240 | 107 | 2705360 | 2.07E-28 |
| Mms22 | 13 | 21240 | 0 | 2705360 | 3.91E-28 |
| Mnn4 | 13 | 21240 | 0 | 2705360 | 3.91E-28 |
| Myo3 | 23 | 21240 | 73 | 2705360 | 1.61E-27 |
| Oms1 | 15 | 21240 | 9 | 2705360 | 3.85E-27 |
| YJL225C | 23 | 21240 | 82 | 2705360 | 1.54E-26 |
| Uso1 | 26 | 21240 | 131 | 2705360 | 1.97E-26 |
| YIL177C | 23 | 21240 | 84 | 2705360 | 2.47E-26 |
| Fmp27 | 15 | 21240 | 11 | 2705360 | 2.91E-26 |
| Dna2 | 12 | 21240 | 0 | 2705360 | 5.01E-26 |
| Mdm1 | 12 | 21240 | 0 | 2705360 | 5.01E-26 |
| YHR080C | 12 | 21240 | 0 | 2705360 | 5.01E-26 |
| YOR019W | 12 | 21240 | 0 | 2705360 | 5.01E-26 |
| Spp41 | 19 | 21240 | 39 | 2705360 | 6.22E-26 |
| Ena1 | 27 | 21240 | 160 | 2705360 | 1.00E-25 |
| Irc20 | 18 | 21240 | 31 | 2705360 | 1.03E-25 |
| Dyn1 | 14 | 21240 | 6 | 2705360 | 1.13E-25 |
| Tcb2 | 26 | 21240 | 152 | 2705360 | 5.77E-25 |
| Yrf1-3 | 22 | 21240 | 89 | 2705360 | 2.03E-24 |
| Yrf1-6 | 22 | 21240 | 89 | 2705360 | 2.03E-24 |
| Yrf1-7 | 22 | 21240 | 89 | 2705360 | 2.03E-24 |
| Cin1 | 11 | 21240 | 0 | 2705360 | 6.43E-24 |
| Mtc5 | 11 | 21240 | 0 | 2705360 | 6.43E-24 |
| Sln1 | 11 | 21240 | 0 | 2705360 | 6.43E-24 |
| Tel1 | 11 | 21240 | 0 | 2705360 | 6.43E-24 |
| YOR338W | 11 | 21240 | 0 | 2705360 | 6.43E-24 |
| Zip2 | 11 | 21240 | 0 | 2705360 | 6.43E-24 |
| Smd1 | 19 | 21240 | 60 | 2705360 | 4.96E-23 |
| Mlp2 | 16 | 21240 | 34 | 2705360 | 7.09E-22 |
| Cdc20 | 10 | 21240 | 0 | 2705360 | 8.25E-22 |
| Dsf2 | 10 | 21240 | 0 | 2705360 | 8.25E-22 |
| Nip100 | 10 | 21240 | 0 | 2705360 | 8.25E-22 |
| Sgs1 | 10 | 21240 | 0 | 2705360 | 8.25E-22 |
| YJR039W | 10 | 21240 | 0 | 2705360 | 8.25E-22 |
| YNL181W | 15 | 21240 | 27 | 2705360 | 1.92E-21 |
| Rsm24 | 15 | 21240 | 28 | 2705360 | 2.93E-21 |
| Cdc24 | 11 | 21240 | 5 | 2705360 | 2.71E-20 |
| YPR097W | 16 | 21240 | 46 | 2705360 | 3.61E-20 |
| YML133C | 18 | 21240 | 74 | 2705360 | 3.82E-20 |
| Nnf2 | 12 | 21240 | 11 | 2705360 | 6.26E-20 |
| Dbf4 | 9 | 21240 | 0 | 2705360 | 1.06E-19 |
| Smc6 | 9 | 21240 | 0 | 2705360 | 1.06E-19 |
| Alk2 | 9 | 21240 | 0 | 2705360 | 1.06E-19 |
| Arn2 | 9 | 21240 | 0 | 2705360 | 1.06E-19 |
| Bud5 | 9 | 21240 | 0 | 2705360 | 1.06E-19 |
| Cst9 | 9 | 21240 | 0 | 2705360 | 1.06E-19 |
| Pol32 | 9 | 21240 | 0 | 2705360 | 1.06E-19 |
| Prp5 | 9 | 21240 | 0 | 2705360 | 1.06E-19 |
| Rrt12 | 9 | 21240 | 0 | 2705360 | 1.06E-19 |
| YER071C | 9 | 21240 | 0 | 2705360 | 1.06E-19 |
| YER184C | 9 | 21240 | 0 | 2705360 | 1.06E-19 |
| YJL206C | 9 | 21240 | 0 | 2705360 | 1.06E-19 |
| YKL050C | 9 | 21240 | 0 | 2705360 | 1.06E-19 |
| YOR296W | 9 | 21240 | 0 | 2705360 | 1.06E-19 |
| YPL141C | 9 | 21240 | 0 | 2705360 | 1.06E-19 |
| Mal12 | 9 | 21240 | 0 | 2705360 | 1.06E-19 |
| Mal32 | 9 | 21240 | 0 | 2705360 | 1.06E-19 |
| Mon1 | 9 | 21240 | 0 | 2705360 | 1.06E-19 |
| Snf2 | 15 | 21240 | 39 | 2705360 | 1.54E-19 |
| Kar3 | 11 | 21240 | 7 | 2705360 | 1.95E-19 |
| Bub3 | 19 | 21240 | 110 | 2705360 | 9.98E-19 |
| Esc2 | 9 | 21240 | 1 | 2705360 | 1.05E-18 |
| Ubr1 | 16 | 21240 | 60 | 2705360 | 1.29E-18 |
| Mlp1 | 22 | 21240 | 180 | 2705360 | 1.53E-18 |
| Ngg1 | 21 | 21240 | 158 | 2705360 | 1.83E-18 |
| Sgf73 | 21 | 21240 | 160 | 2705360 | 2.44E-18 |
| Gcv2 | 31 | 21240 | 471 | 2705360 | 3.13E-18 |
| Stu1 | 9 | 21240 | 2 | 2705360 | 5.74E-18 |
| Aos1 | 19 | 21240 | 122 | 2705360 | 5.60E-18 |
| Sok1 | 9 | 21240 | 2 | 2705360 | 5.74E-18 |
| YMR102C | 9 | 21240 | 2 | 2705360 | 5.74E-18 |
| Spa2 | 22 | 21240 | 196 | 2705360 | 7.97E-18 |
| Acm1 | 8 | 21240 | 0 | 2705360 | 1.36E-17 |
| Nbp1 | 8 | 21240 | 0 | 2705360 | 1.36E-17 |
| Aad14 | 8 | 21240 | 0 | 2705360 | 1.36E-17 |
| Aep3 | 8 | 21240 | 0 | 2705360 | 1.36E-17 |
| Aim22 | 8 | 21240 | 0 | 2705360 | 1.36E-17 |
| Fun19 | 8 | 21240 | 0 | 2705360 | 1.36E-17 |
| Pes4 | 8 | 21240 | 0 | 2705360 | 1.36E-17 |
| Prp28 | 8 | 21240 | 0 | 2705360 | 1.36E-17 |
| Rtc1 | 8 | 21240 | 0 | 2705360 | 1.36E-17 |
| Sas5 | 8 | 21240 | 0 | 2705360 | 1.36E-17 |
| Slz1 | 8 | 21240 | 0 | 2705360 | 1.36E-17 |
| Stb2 | 8 | 21240 | 0 | 2705360 | 1.36E-17 |
| Tad1 | 8 | 21240 | 0 | 2705360 | 1.36E-17 |
| Tos4 | 8 | 21240 | 0 | 2705360 | 1.36E-17 |
| YBL107C | 8 | 21240 | 0 | 2705360 | 1.36E-17 |
| YDR222W | 8 | 21240 | 0 | 2705360 | 1.36E-17 |
| YDR282C | 8 | 21240 | 0 | 2705360 | 1.36E-17 |
| YGR079W | 8 | 21240 | 0 | 2705360 | 1.36E-17 |
| Yhp1 | 8 | 21240 | 0 | 2705360 | 1.36E-17 |
| YPL150W | 8 | 21240 | 0 | 2705360 | 1.36E-17 |
| Ama1 | 7 | 21240 | 0 | 2705360 | 1.74E-17 |
| Spc97 | 9 | 21240 | 3 | 2705360 | 2.28E-17 |
| Gin4 | 15 | 21240 | 58 | 2705360 | 2.26E-17 |
| Sip2 | 21 | 21240 | 188 | 2705360 | 4.80E-17 |
| Sak1 | 9 | 21240 | 4 | 2705360 | 7.36E-17 |
| Nam2 | 9 | 21240 | 6 | 2705360 | 7.36E-17 |
| Rib2 | 9 | 21240 | 6 | 2705360 | 7.36E-17 |
| Hop1 | 8 | 21240 | 1 | 2705360 | 1.21E-16 |
| Gsm1 | 8 | 21240 | 1 | 2705360 | 1.21E-16 |
| Iml1 | 8 | 21240 | 1 | 2705360 | 1.21E-16 |
| Shy1 | 8 | 21240 | 1 | 2705360 | 1.21E-16 |
| YJR141W | 8 | 21240 | 1 | 2705360 | 1.21E-16 |
| Dam1 | 10 | 21240 | 10 | 2705360 | 1.42E-16 |
| Smc4 | 12 | 21240 | 27 | 2705360 | 1.61E-16 |
| Ser33 | 55 | 21240 | 1816 | 2705360 | 3.33E-16 |
| Yat2 | 9 | 21240 | 6 | 2705360 | 5.08E-16 |
| Msh1 | 8 | 21240 | 2 | 2705360 | 6.03E-16 |
| Sap1 | 8 | 21240 | 2 | 2705360 | 6.03E-16 |
| Sps1 | 8 | 21240 | 2 | 2705360 | 6.03E-16 |
| Vac7 | 8 | 21240 | 2 | 2705360 | 6.03E-16 |
| Pnp1 | 17 | 21240 | 120 | 2705360 | 1.25E-15 |
| Pog1 | 7 | 21240 | 0 | 2705360 | 1.74E-15 |
| Sfi1 | 7 | 21240 | 0 | 2705360 | 1.74E-15 |
| Sli15 | 7 | 21240 | 0 | 2705360 | 1.74E-15 |
| Rad9 | 7 | 21240 | 0 | 2705360 | 1.74E-15 |
| Afr1 | 7 | 21240 | 0 | 2705360 | 1.74E-15 |
| Aim44 | 7 | 21240 | 0 | 2705360 | 1.74E-15 |
| Atg7 | 7 | 21240 | 0 | 2705360 | 1.74E-15 |
| Bit2 | 7 | 21240 | 0 | 2705360 | 1.74E-15 |
| Bnr1 | 7 | 21240 | 0 | 2705360 | 1.74E-15 |
| Bud8 | 7 | 21240 | 0 | 2705360 | 1.74E-15 |
| Bud9 | 7 | 21240 | 0 | 2705360 | 1.74E-15 |
| Cwc24 | 7 | 21240 | 0 | 2705360 | 1.74E-15 |
| Don1 | 7 | 21240 | 0 | 2705360 | 1.74E-15 |
| Eki1 | 7 | 21240 | 0 | 2705360 | 1.74E-15 |
| Fre6 | 7 | 21240 | 0 | 2705360 | 1.74E-15 |
| Gpi10 | 7 | 21240 | 0 | 2705360 | 1.74E-15 |
| Itr2 | 7 | 21240 | 0 | 2705360 | 1.74E-15 |
| Mcm10 | 7 | 21240 | 0 | 2705360 | 1.74E-15 |
| Opt2 | 7 | 21240 | 0 | 2705360 | 1.74E-15 |
| Pkh3 | 7 | 21240 | 0 | 2705360 | 1.74E-15 |
| Sal1 | 7 | 21240 | 0 | 2705360 | 1.74E-15 |
| Thi22 | 7 | 21240 | 0 | 2705360 | 1.74E-15 |
| Tus1 | 7 | 21240 | 0 | 2705360 | 1.74E-15 |
| Vps30 | 7 | 21240 | 0 | 2705360 | 1.74E-15 |
| YBR225W | 7 | 21240 | 0 | 2705360 | 1.74E-15 |
| YIL092W | 7 | 21240 | 0 | 2705360 | 1.74E-15 |
| YJR061W | 7 | 21240 | 0 | 2705360 | 1.74E-15 |
| YLR455W | 7 | 21240 | 0 | 2705360 | 1.74E-15 |
| YPL216W | 7 | 21240 | 0 | 2705360 | 1.74E-15 |
| Fyv8 | 9 | 21240 | 9 | 2705360 | 4.83E-15 |
| Mph1 | 9 | 21240 | 9 | 2705360 | 4.83E-15 |
| Mtf2 | 9 | 21240 | 9 | 2705360 | 4.83E-15 |
| Ism1 | 8 | 21240 | 4 | 2705360 | 6.54E-15 |
| Suv3 | 8 | 21240 | 4 | 2705360 | 6.54E-15 |
| YFR016C | 16 | 21240 | 114 | 2705360 | 9.72E-15 |
| Bdf2 | 7 | 21240 | 1 | 2705360 | 9.72E-15 |
| Aro80 | 7 | 21240 | 1 | 2705360 | 1.38E-14 |
| Iba57 | 7 | 21240 | 1 | 2705360 | 1.38E-14 |
| Sac3 | 7 | 21240 | 1 | 2705360 | 1.38E-14 |
| Taz1 | 7 | 21240 | 1 | 2705360 | 1.38E-14 |
| Ubp7 | 7 | 21240 | 1 | 2705360 | 1.38E-14 |
| Vps36 | 7 | 21240 | 1 | 2705360 | 1.38E-14 |
| Bud4 | 9 | 21240 | 11 | 2705360 | 1.65E-14 |
| Fus1 | 9 | 21240 | 11 | 2705360 | 1.65E-14 |
| Src1 | 9 | 21240 | 11 | 2705360 | 1.65E-14 |
| Rad50 | 13 | 21240 | 60 | 2705360 | 2.18E-14 |
| Spc42 | 8 | 21240 | 6 | 2705360 | 3.91E-14 |
| Prp4 | 8 | 21240 | 6 | 2705360 | 3.91E-14 |
| Fmp10 | 7 | 21240 | 2 | 2705360 | 6.19E-14 |
| Gpb1 | 7 | 21240 | 2 | 2705360 | 6.19E-14 |
| Msl5 | 7 | 21240 | 2 | 2705360 | 6.19E-14 |
| Nat3 | 7 | 21240 | 2 | 2705360 | 6.19E-14 |
| Spt23 | 7 | 21240 | 2 | 2705360 | 6.19E-14 |
| Sun4 | 7 | 21240 | 2 | 2705360 | 6.19E-14 |
| Upc2 | 7 | 21240 | 2 | 2705360 | 6.19E-14 |
| Aim23 | 8 | 21240 | 7 | 2705360 | 8.33E-14 |
| Bsp1 | 8 | 21240 | 7 | 2705360 | 8.33E-14 |
| Fab1 | 8 | 21240 | 7 | 2705360 | 8.33E-14 |
| Brn1 | 11 | 21240 | 37 | 2705360 | 1.11E-13 |
| Rad26 | 8 | 21240 | 8 | 2705360 | 1.11E-13 |
| Pep5 | 8 | 21240 | 8 | 2705360 | 1.65E-13 |
| Top2 | 20 | 21240 | 261 | 2705360 | 1.90E-13 |
| Aft1 | 7 | 21240 | 3 | 2705360 | 2.05E-13 |
| Cyk3 | 7 | 21240 | 3 | 2705360 | 2.05E-13 |
| Pmd1 | 7 | 21240 | 3 | 2705360 | 2.05E-13 |
| Red1 | 7 | 21240 | 3 | 2705360 | 2.05E-13 |
| Slu7 | 7 | 21240 | 3 | 2705360 | 2.05E-13 |
| Siz1 | 6 | 21240 | 0 | 2705360 | 2.24E-13 |
| Rad34 | 6 | 21240 | 0 | 2705360 | 2.24E-13 |
| Rad54 | 6 | 21240 | 0 | 2705360 | 2.24E-13 |
| Rad61 | 6 | 21240 | 0 | 2705360 | 2.24E-13 |
| Aad6 | 6 | 21240 | 0 | 2705360 | 2.24E-13 |
| Adk2 | 6 | 21240 | 0 | 2705360 | 2.24E-13 |
| Asi1 | 6 | 21240 | 0 | 2705360 | 2.24E-13 |
| Bre4 | 6 | 21240 | 0 | 2705360 | 2.24E-13 |
| Cos111 | 6 | 21240 | 0 | 2705360 | 2.24E-13 |
| Ctl1 | 6 | 21240 | 0 | 2705360 | 2.24E-13 |
| Eds1 | 6 | 21240 | 0 | 2705360 | 2.24E-13 |
| Flo8 | 6 | 21240 | 0 | 2705360 | 2.24E-13 |
| Fus2 | 6 | 21240 | 0 | 2705360 | 2.24E-13 |
| Fyv6 | 6 | 21240 | 0 | 2705360 | 2.24E-13 |
| Gip2 | 6 | 21240 | 0 | 2705360 | 2.24E-13 |
| Hrq1 | 6 | 21240 | 0 | 2705360 | 2.24E-13 |
| Isa1 | 6 | 21240 | 0 | 2705360 | 2.24E-13 |
| Ldb19 | 6 | 21240 | 0 | 2705360 | 2.24E-13 |
| Mak32 | 6 | 21240 | 0 | 2705360 | 2.24E-13 |
| Met32 | 6 | 21240 | 0 | 2705360 | 2.24E-13 |
| Mmt2 | 6 | 21240 | 0 | 2705360 | 2.24E-13 |
| Npr3 | 6 | 21240 | 0 | 2705360 | 2.24E-13 |
| Prk1 | 6 | 21240 | 0 | 2705360 | 2.24E-13 |
| Q0255 | 6 | 21240 | 0 | 2705360 | 2.24E-13 |
| Rfx1 | 6 | 21240 | 0 | 2705360 | 2.24E-13 |
| Rrm3 | 6 | 21240 | 0 | 2705360 | 2.24E-13 |
| Rts3 | 6 | 21240 | 0 | 2705360 | 2.24E-13 |
| Set4 | 6 | 21240 | 0 | 2705360 | 2.24E-13 |
| Spp2 | 6 | 21240 | 0 | 2705360 | 2.24E-13 |
| Spr3 | 6 | 21240 | 0 | 2705360 | 2.24E-13 |
| Srs2 | 6 | 21240 | 0 | 2705360 | 2.24E-13 |
| Tam41 | 6 | 21240 | 0 | 2705360 | 2.24E-13 |
| Ubp11 | 6 | 21240 | 0 | 2705360 | 2.24E-13 |
| Urc2 | 6 | 21240 | 0 | 2705360 | 2.24E-13 |
| Vps38 | 6 | 21240 | 0 | 2705360 | 2.24E-13 |
| Vps70 | 6 | 21240 | 0 | 2705360 | 2.24E-13 |
| YDL133W | 6 | 21240 | 0 | 2705360 | 2.24E-13 |
| YDR109C | 6 | 21240 | 0 | 2705360 | 2.24E-13 |
| YDR455C | 6 | 21240 | 0 | 2705360 | 2.24E-13 |
| YEL023C | 6 | 21240 | 0 | 2705360 | 2.24E-13 |
| YGR053C | 6 | 21240 | 0 | 2705360 | 2.24E-13 |
| YMR185W | 6 | 21240 | 0 | 2705360 | 2.24E-13 |
| YOL075C | 6 | 21240 | 0 | 2705360 | 2.24E-13 |
| Yox1 | 6 | 21240 | 0 | 2705360 | 2.24E-13 |
| YPR157W | 6 | 21240 | 0 | 2705360 | 2.24E-13 |
| YKR078W | 6 | 21240 | 0 | 2705360 | 2.24E-13 |
| Jhd1 | 6 | 21240 | 0 | 2705360 | 2.24E-13 |
| Mgr1 | 8 | 21240 | 9 | 2705360 | 3.10E-13 |
| Sbe22 | 8 | 21240 | 9 | 2705360 | 3.10E-13 |
| Nup159 | 18 | 21240 | 207 | 2705360 | 4.13E-13 |
| Rad5 | 7 | 21240 | 4 | 2705360 | 5.60E-13 |
| Atg26 | 7 | 21240 | 4 | 2705360 | 5.60E-13 |
| Mnr2 | 7 | 21240 | 4 | 2705360 | 5.60E-13 |
| Prp2 | 7 | 21240 | 4 | 2705360 | 5.60E-13 |
| Ubr2 | 7 | 21240 | 4 | 2705360 | 5.60E-13 |
| Bni4 | 7 | 21240 | 5 | 2705360 | 1.33E-12 |
| Set1 | 7 | 21240 | 5 | 2705360 | 1.33E-12 |
| Drs1 | 18 | 21240 | 225 | 2705360 | 1.52E-12 |
| Dus4 | 6 | 21240 | 1 | 2705360 | 1.56E-12 |
| Icl2 | 6 | 21240 | 1 | 2705360 | 1.56E-12 |
| Mrpl16 | 6 | 21240 | 1 | 2705360 | 1.56E-12 |
| Rom2 | 6 | 21240 | 1 | 2705360 | 1.56E-12 |
| Vps3 | 6 | 21240 | 1 | 2705360 | 1.56E-12 |
| YOR022C | 6 | 21240 | 1 | 2705360 | 1.56E-12 |
| YOR093C | 6 | 21240 | 1 | 2705360 | 1.56E-12 |
| Kog1 | 8 | 21240 | 13 | 2705360 | 2.53E-12 |
| Pkh2 | 8 | 21240 | 13 | 2705360 | 2.53E-12 |
| Cdc25 | 7 | 21240 | 6 | 2705360 | 2.87E-12 |
| Iqg1 | 7 | 21240 | 6 | 2705360 | 2.87E-12 |
| Ski7 | 7 | 21240 | 6 | 2705360 | 2.87E-12 |
| Usa1 | 7 | 21240 | 6 | 2705360 | 2.87E-12 |
| Mip1 | 8 | 21240 | 14 | 2705360 | 3.94E-12 |
| Air1 | 7 | 21240 | 7 | 2705360 | 5.70E-12 |
| Set3 | 7 | 21240 | 7 | 2705360 | 5.70E-12 |
| Upf3 | 7 | 21240 | 7 | 2705360 | 5.70E-12 |
| Set5 | 8 | 21240 | 15 | 2705360 | 6.00E-12 |
| Bre2 | 6 | 21240 | 2 | 2705360 | 6.18E-12 |
| Mrp21 | 6 | 21240 | 2 | 2705360 | 6.18E-12 |
| Rim2 | 6 | 21240 | 2 | 2705360 | 6.18E-12 |
| Pac10 | 17 | 21240 | 213 | 2705360 | 6.49E-12 |
| Cbp4 | 8 | 21240 | 16 | 2705360 | 8.94E-12 |
| Blm10 | 11 | 21240 | 60 | 2705360 | 1.07E-11 |
| Dia4 | 5 | 21240 | 1 | 2705360 | 1.71E-11 |
| Hul4 | 5 | 21240 | 1 | 2705360 | 1.71E-11 |
| Mms1 | 5 | 21240 | 1 | 2705360 | 1.71E-11 |
| Nst1 | 5 | 21240 | 1 | 2705360 | 1.71E-11 |
| Prp40 | 5 | 21240 | 1 | 2705360 | 1.71E-11 |
| Ptp1 | 5 | 21240 | 1 | 2705360 | 1.71E-11 |
| Sbe2 | 5 | 21240 | 1 | 2705360 | 1.71E-11 |
| Slx4 | 5 | 21240 | 1 | 2705360 | 1.71E-11 |
| Ulp2 | 5 | 21240 | 1 | 2705360 | 1.71E-11 |
| Caf130 | 6 | 21240 | 3 | 2705360 | 1.84E-11 |
| Rga1 | 6 | 21240 | 3 | 2705360 | 1.84E-11 |
| Rnt1 | 6 | 21240 | 3 | 2705360 | 1.84E-11 |
| Snu66 | 6 | 21240 | 3 | 2705360 | 1.84E-11 |
| Tpo1 | 6 | 21240 | 3 | 2705360 | 1.84E-11 |
| YLL032C | 6 | 21240 | 3 | 2705360 | 1.84E-11 |
| Vps17 | 7 | 21240 | 9 | 2705360 | 1.87E-11 |
| Lys9 | 47 | 21240 | 1879 | 2705360 | 2.57E-11 |
| Nup133 | 15 | 21240 | 169 | 2705360 | 2.75E-11 |
| Gis4 | 5 | 21240 | 0 | 2705360 | 2.87E-11 |
| Esp1 | 5 | 21240 | 0 | 2705360 | 2.87E-11 |
| Mad2 | 5 | 21240 | 0 | 2705360 | 2.87E-11 |
| Pds1 | 5 | 21240 | 0 | 2705360 | 2.87E-11 |
| Esc8 | 5 | 21240 | 0 | 2705360 | 2.87E-11 |
| Pzf1 | 5 | 21240 | 0 | 2705360 | 2.87E-11 |
| Atp25 | 5 | 21240 | 0 | 2705360 | 2.87E-11 |
| Bbp1 | 5 | 21240 | 0 | 2705360 | 2.87E-11 |
| Cax4 | 5 | 21240 | 0 | 2705360 | 2.87E-11 |
| Cbs2 | 5 | 21240 | 0 | 2705360 | 2.87E-11 |
| Cox10 | 5 | 21240 | 0 | 2705360 | 2.87E-11 |
| Csr2 | 5 | 21240 | 0 | 2705360 | 2.87E-11 |
| Din7 | 5 | 21240 | 0 | 2705360 | 2.87E-11 |
| Ecm3 | 5 | 21240 | 0 | 2705360 | 2.87E-11 |
| Eco1 | 5 | 21240 | 0 | 2705360 | 2.87E-11 |
| Elm1 | 5 | 21240 | 0 | 2705360 | 2.87E-11 |
| Fdh1 | 5 | 21240 | 0 | 2705360 | 2.87E-11 |
| Hap4 | 5 | 21240 | 0 | 2705360 | 2.87E-11 |
| Hmi1 | 5 | 21240 | 0 | 2705360 | 2.87E-11 |
| Rec107 | 5 | 21240 | 0 | 2705360 | 2.87E-11 |
| Cos5 | 5 | 21240 | 0 | 2705360 | 2.87E-11 |
| Kin82 | 5 | 21240 | 0 | 2705360 | 2.87E-11 |
| Ktr2 | 5 | 21240 | 0 | 2705360 | 2.87E-11 |
| Mal33 | 5 | 21240 | 0 | 2705360 | 2.87E-11 |
| Mlh2 | 5 | 21240 | 0 | 2705360 | 2.87E-11 |
| Mth1 | 5 | 21240 | 0 | 2705360 | 2.87E-11 |
| Nha1 | 5 | 21240 | 0 | 2705360 | 2.87E-11 |
| Pet122 | 5 | 21240 | 0 | 2705360 | 2.87E-11 |
| Plp1 | 5 | 21240 | 0 | 2705360 | 2.87E-11 |
| Prp22 | 5 | 21240 | 0 | 2705360 | 2.87E-11 |
| Ptp2 | 5 | 21240 | 0 | 2705360 | 2.87E-11 |
| Pxl1 | 5 | 21240 | 0 | 2705360 | 2.87E-11 |
| Rft1 | 5 | 21240 | 0 | 2705360 | 2.87E-11 |
| Rmd5 | 5 | 21240 | 0 | 2705360 | 2.87E-11 |
| Rri2 | 5 | 21240 | 0 | 2705360 | 2.87E-11 |
| Rtc4 | 5 | 21240 | 0 | 2705360 | 2.87E-11 |
| Sld3 | 5 | 21240 | 0 | 2705360 | 2.87E-11 |
| Spo22 | 5 | 21240 | 0 | 2705360 | 2.87E-11 |
| Spo75 | 5 | 21240 | 0 | 2705360 | 2.87E-11 |
| Spp382 | 5 | 21240 | 0 | 2705360 | 2.87E-11 |
| Sps2 | 5 | 21240 | 0 | 2705360 | 2.87E-11 |
| Sps4 | 5 | 21240 | 0 | 2705360 | 2.87E-11 |
| Spt21 | 5 | 21240 | 0 | 2705360 | 2.87E-11 |
| Ssk22 | 5 | 21240 | 0 | 2705360 | 2.87E-11 |
| Syf2 | 5 | 21240 | 0 | 2705360 | 2.87E-11 |
| Tgs1 | 5 | 21240 | 0 | 2705360 | 2.87E-11 |
| Tos2 | 5 | 21240 | 0 | 2705360 | 2.87E-11 |
| Trk1 | 5 | 21240 | 0 | 2705360 | 2.87E-11 |
| Yap7 | 5 | 21240 | 0 | 2705360 | 2.87E-11 |
| YDR357C | 5 | 21240 | 0 | 2705360 | 2.87E-11 |
| YDR514C | 5 | 21240 | 0 | 2705360 | 2.87E-11 |
| YER128W | 5 | 21240 | 0 | 2705360 | 2.87E-11 |
| YER186C | 5 | 21240 | 0 | 2705360 | 2.87E-11 |
| YIR024C | 5 | 21240 | 0 | 2705360 | 2.87E-11 |
| YJL049W | 5 | 21240 | 0 | 2705360 | 2.87E-11 |
| YJR030C | 5 | 21240 | 0 | 2705360 | 2.87E-11 |
| YJR098C | 5 | 21240 | 0 | 2705360 | 2.87E-11 |
| YKR017C | 5 | 21240 | 0 | 2705360 | 2.87E-11 |
| YLR049C | 5 | 21240 | 0 | 2705360 | 2.87E-11 |
| YLR218C | 5 | 21240 | 0 | 2705360 | 2.87E-11 |
| YMR124W | 5 | 21240 | 0 | 2705360 | 2.87E-11 |
| Ynd1 | 5 | 21240 | 0 | 2705360 | 2.87E-11 |
| YNR063W | 5 | 21240 | 0 | 2705360 | 2.87E-11 |
| YOL019W-A | 5 | 21240 | 0 | 2705360 | 2.87E-11 |
| YOR102W | 5 | 21240 | 0 | 2705360 | 2.87E-11 |
| Ysp1 | 5 | 21240 | 0 | 2705360 | 2.87E-11 |
| SAT4 | 5 | 21240 | 0 | 2705360 | 2.87E-11 |
| Rrn11 | 7 | 21240 | 10 | 2705360 | 3.17E-11 |
| Rsm26 | 7 | 21240 | 10 | 2705360 | 3.17E-11 |
| Tao3 | 8 | 21240 | 20 | 2705360 | 3.67E-11 |
| Pex6 | 6 | 21240 | 4 | 2705360 | 4.57E-11 |
| Prp16 | 6 | 21240 | 4 | 2705360 | 4.57E-11 |
| Psd2 | 6 | 21240 | 4 | 2705360 | 4.57E-11 |
| Slp1 | 6 | 21240 | 4 | 2705360 | 4.57E-11 |
| Smb1 | 6 | 21240 | 4 | 2705360 | 4.57E-11 |
| Ybp1 | 6 | 21240 | 4 | 2705360 | 4.57E-11 |
| Pol4 | 7 | 21240 | 11 | 2705360 | 5.15E-11 |
| Laa1 | 8 | 21240 | 22 | 2705360 | 6.82E-11 |
| Stb5 | 8 | 21240 | 22 | 2705360 | 6.82E-11 |
| Sua5 | 8 | 21240 | 22 | 2705360 | 6.82E-11 |
| Brr2 | 7 | 21240 | 12 | 2705360 | 8.09E-11 |
| Sec2 | 7 | 21240 | 12 | 2705360 | 8.09E-11 |
| Mtr4 | 19 | 21240 | 331 | 2705360 | 8.20E-11 |
| Bul2 | 8 | 21240 | 23 | 2705360 | 9.10E-11 |
| Abc1 | 6 | 21240 | 5 | 2705360 | 9.99E-11 |
| Fyv7 | 6 | 21240 | 5 | 2705360 | 9.99E-11 |
| Ups2 | 6 | 21240 | 5 | 2705360 | 9.99E-11 |

Only those proteins with p < 1.00E-10 are listed. * Number indicates the sum of all peptides corresponding to proteins found at peptide spectrum counts of 3 or more in the Slk9-TAP sample. ** Number indicates the sum of all peptides in Peptide Atlas corresponding to proteins found at peptide spectrum counts of 3 or more in the Slk19-TAP sample. # P-value according to Fisher’s Exact Test.
